# Benchmarking functional connectivity by the structure and geometry of the human brain

**DOI:** 10.1162/netn_a_00236

**Published:** 2022-10-01

**Authors:** Zhen-Qi Liu, Richard F. Betzel, Bratislav Misic

**Affiliations:** McConnell Brain Imaging Centre, Montréal Neurological Institute, McGill University, Montréal, Canada; Psychological and Brain Sciences, Indiana University, Bloomington, IN, USA

**Keywords:** Connectome, Functional connectivity, Structural connectivity, Geometry, Embedding, Gradient, Hierarchy, Transmodal

## Abstract

The brain’s structural connectivity supports the propagation of electrical impulses, manifesting as patterns of coactivation, termed functional connectivity. Functional connectivity emerges from the underlying sparse structural connections, particularly through polysynaptic communication. As a result, functional connections between brain regions without direct structural links are numerous, but their organization is not completely understood. Here we investigate the organization of functional connections without direct structural links. We develop a simple, data-driven method to benchmark functional connections with respect to their underlying structural and geometric embedding. We then use this method to reweigh and reexpress functional connectivity. We find evidence of unexpectedly strong functional connectivity among distal brain regions and within the default mode network. We also find unexpectedly strong functional connectivity at the apex of the unimodal-transmodal hierarchy. Our results suggest that both phenomena—functional modules and functional hierarchies—emerge from functional interactions that transcend the underlying structure and geometry. These findings also potentially explain recent reports that structural and functional connectivity gradually diverge in transmodal cortex. Collectively, we show how structural connectivity and geometry can be used as a natural frame of reference with which to study functional connectivity patterns in the brain.

## Author Summary

The structural connectivity of the brain supports interregional signaling, manifesting as highly organized patterns of functional connectivity. Importantly, structural and functional connectivity are fundamentally constrained by the spatial embedding of brain regions, such that proximal regions are more likely to exhibit stronger connectivity. Here we develop a simple method that uses robust relationships between geometry, structure, and function as the baseline to reweigh and reexpress functional connectivity. We use the method to identify functional connections that are greater than expected given their structural and geometric embedding. We then show that the arrangement of these connections systematically follows the functional modules and the putative unimodal-transmodal hierarchy of the brain. Collectively, our findings demonstrate highly organized patterns of polysynaptic functional connections that support the emergence of canonical features of functional connectivity networks, including modules and hierarchies.

## INTRODUCTION

Axonal wiring among neurons and neuronal populations promotes signal exchange and information integration. At the mesoscale, signaling via the complex network of structural connectivity (SC) manifests as patterns of temporal correlations, termed functional connectivity (FC). Functional connectivity is highly organized (Bellec, Rosa-Neto, Lyttelton, Benali, & Evans, 2010; Damoiseaux et al., 2006; Yeo et al., 2011), reproducible (Gordon et al., 2017; Noble, Scheinost, & Constable, 2019), and related to individual differences in behaviour (Mišić & Sporns, 2016; Smith et al., 2015).

**Figure 1.**
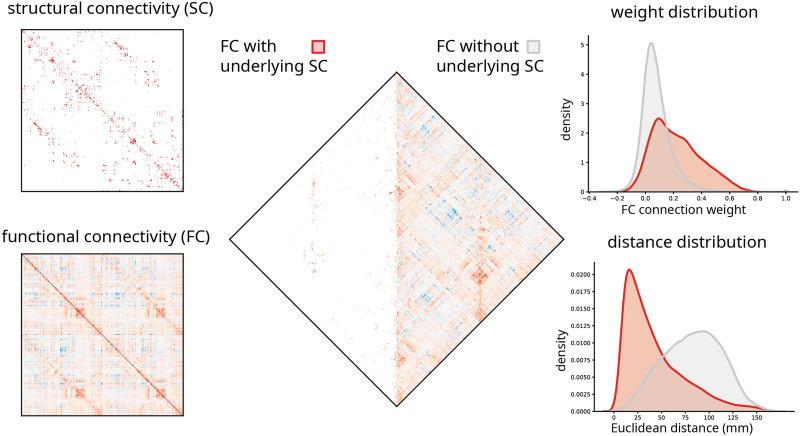
Functional connections with and without direct structural links. Left: Structural connectivity (SC) and functional connectivity (FC) matrices in the 1,000-node Lausanne parcellation (Cammoun et al., 2012). Middle: Functional connections with and without underlying structural connections. Right: The weight and anatomical (Euclidean) distance distribution of the two types of functional connections.

Most pairwise functional connections are not supported by a direct structural connection. By definition, functional networks are fully connected, while structural networks are sparse (Figure 1). Across species, reconstruction techniques, and spatial scales, structural connection density is typically reported to be between 2% and 40% (Van den Heuvel, Bullmore, & Sporns, 2016) (but see also Markov et al., 2013), meaning that the majority of functional connections between two regions are not accompanied by a corresponding monosynaptic structural connection. These “indirect” functional connections are thought to emerge from polysynaptic communication in the structural network (Bazinet, Vos de Wael, Hagmann, Bernhardt, & Misic, 2021; Suárez, Markello, Betzel, & Misic, 2020).

Importantly, structural and functional connectivity are fundamentally constrained by the spatial embedding of brain regions (Stiso & Bassett, 2018). Structural connection probability is inversely correlated with spatial separation, such that proxmimal neural elements are more likely to be structurally connected, while distant neural elements are less likely to be connected (Horvát et al., 2016; Markov et al., 2013; Roberts et al., 2016). A similar distance dependence is also observed for functional connectivity (Margulies et al., 2016; Mišić et al., 2014; Salvador et al., 2005; Sepulcre et al., 2010). The overrepresentation of low-cost, short-range connections is thought to reflect finite material and metabolic resources (Figure 1) (Bullmore & Sporns, 2012). Altogether, structural connectivity and spatial proximity constitute a natural frame of reference for understanding and interpreting functional connectivity.

Here we investigate the organization of functional connections without direct structural links (Figure 1). We develop a simple method that uses robust relationships between geometry, structure, and function as the baseline to reweigh and reexpress functional connectivity. We use the method to identify functional connections that are greater than expected given their structural and geometric embedding. We then show that the arrangement of these connections systematically follows the functional modules (intrinsic networks) (Yeo et al., 2011) and the putative unimodal-transmodal hierarchy of the brain (Margulies et al., 2016).

## RESULTS

The results are organized as follows. We first establish a method to quantify how unexpectedly strong a functional connection is given the physical Euclidean distance between its connected areas. We then describe the organizational principles of these structurally unconnected functional connections by characterizing their (1) statistical properties, (2) correspondence with intrinsic networks, and (3) correspondence with the cortical hierarchy. Data sources include (see Materials and Methods for detailed procedures):

▪  *Structural connectivity*. Structural and functional connectivity were derived from *N* = 66 healthy control participants (source: Lausanne University Hospital; https://doi.org/10.5281/zenodo.2872624) using the 1,000-node Lausanne parcellation (Cammoun et al., 2012). Participants were randomly divided into a *Discovery* and *Validation* cohort (*N* = 33 each). Structural connectivity was reconstructed using diffusion spectrum imaging and deterministic streamline tractography. A consistency- and length-based procedure was then used to assemble a group-representative structural connectivity matrix (Betzel, Griffa, Hagmann, & Mišić, 2018; Mišić et al., 2015, 2018).
▪  *Functional connectivity*. Functional connectivity was estimated in the same individuals by using resting-state functional MRI (rs-fMRI). A functional connectivity matrix was constructed using pairwise Pearson correlations among regional time courses. A group-average functional connectivity matrix was then estimated as the mean connectivity of pairwise connections across individuals.

### Long-Range Functional Connections Are Unexpectedly Strong

**Figure 2.**
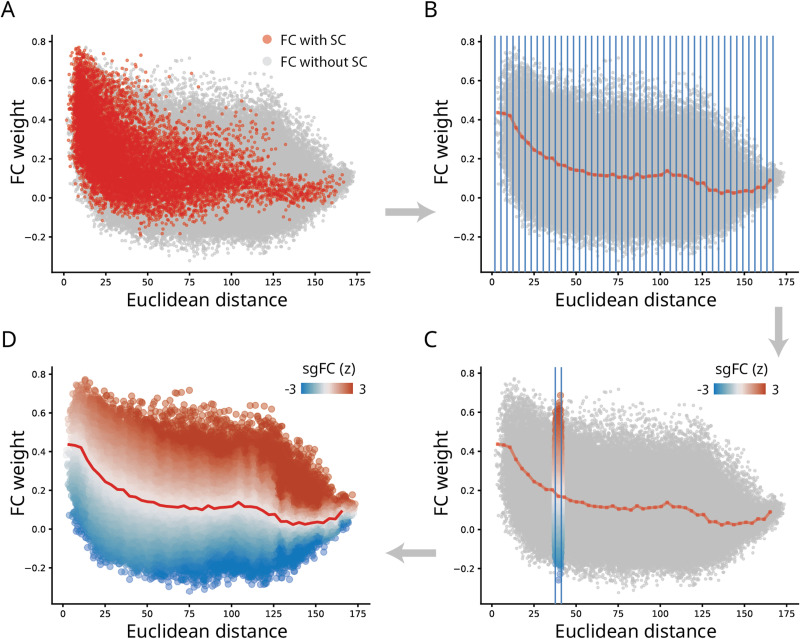
Benchmarking functional connectivity by structure and geometry. (A) FC connection weight-to-distance relationship shown for FC with (red) and without (gray) direct SC connections, respectively. (B) FCs grouped into distances bins (blue lines), and the mean value within each bin of those with direct SCs (dotted red line). (C) Within a sample bin, unconnected (polysynaptic) FCs are expressed as a z-score relative to connected (monosynaptic) FCs. We refer to this *z*-score as structure- and geometry-informed FC (sgFC). (D) sgFCs shown as a smoothly-transitioning spectrum after the procedure is applied for each distance bin. See Methods for more technical details and Supporting Information Figure S2 for details about smoothing and bin size selection.

To quantify how unexpectedly strong a functional connection is, we first seek to establish a baseline (for a conceptually similar approach, see Roberts et al., 2016). Figure 2A shows the relationship between the spatial separation of two nodes (abscissa) and the functional connectivity between them (ordinate). Functional connections that are supported by an underlying structural connection are shown in red, and all other functional connections, which we refer to as indirect or structurally unconnected FCs, are shown in gray. We note the classical exponential decrease in magnitude with increasing spatial separation (Roberts et al., 2016; Stiso & Bassett, 2018). We also note that connected (monosynaptic) and unconnected (polysynaptic) FCs have similar distributions at short distances, but that they diverge considerably at long distances. Namely, when the spatial separation between two regions is greater than approximately 125 mm, there is greater variability among unconnected FCs, with many unconnected FCs marked by greater magnitude than connected FCs spanning comparable distances.

We therefore set the magnitude of connected FCs at a given distance as the baseline for unconnected FCs at a comparable distance. The goal is to identify unconnected FCs that are unexpectedly large relative to connected FCs. To operationalize this intuition, we first bin FCs according to their spatial proximity (Figure 2B). Within each bin, we record the distribution of connected FCs, including their mean and standard deviation. Finally, we express each unconnected FC as a z-score relative to the distribution of connected FCs in the same distance bin (Figure 2C). This measure reflects how unexpectedly strong a functional connection is, given its length. Importantly, z-scores for unconnected FCs are estimated based on moments of a distribution estimated for connected FCs. For simplicity, we term the reexpressed unconnected FCs as structure- and geometry-informed FC (sgFC).

Figure 2D shows the reweighing of unconnected FCs. Across the entire range of distances, there exist many unconnected FCs that are disproportionately strong relative to their length. A population of unconnected positive FCs spanning distances greater than 125 mm are particularly prominent, suggesting the existence of multiple strong functional interactions above and beyond what would be expected on the basis of their length. Values of sgFC have a distribution centered around zero, with a long positive tail (Supporting Information Figure S1). In the following sections we explore the organization of these connections in greater detail. For sensitivity analyses regarding bin sizes, preprocessing choices and validation, please see Control Analyses section and Supporting Information Figures S2 and S3. For replication in individual participants, see Supporting Information Figure S4.

### Contribution to Intrinsic Network Architecture

**Figure 3.**
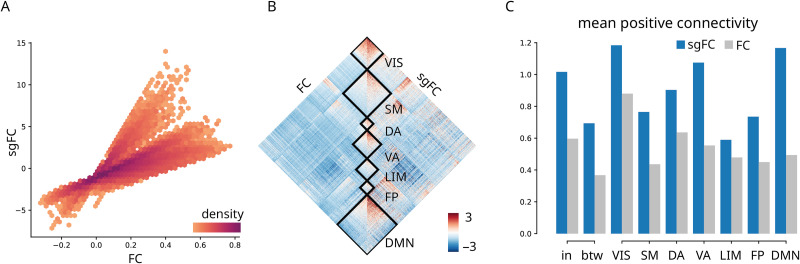
Contribution to intrinsic network architecture. (A) sgFC correlated with FC, colored by scatter density. Only polysynaptic FCs are shown. (B) FC and sgFC shown side-by-side, reordered according intrinsic networks (Yeo et al., 2011). VIS = visual; SM = somatomotor; DA = dorsal attention; VA = ventral attention; LIM = limbic; FP = frontoparietal; DMN = default mode. (C) Comparison of within- and between-network mean positive-valued connectivity with a dissection of within-network connectivity for intrinsic networks. (sgFC to FC ratios: in = 1.70; betw = 1.89; VIS = 1.35; SM = 1.75; DA = 1.42; VA = 1.94; LIM = 1.23; FP = 1.63; DMN = 2.36). In panels B and C, polysynaptic FCs are standardized by the overall average and standard deviation of FCs with direct SCs to facilitate comparison.

We first ask how conventional FC and sgFC are related to each other and how they are distributed within and between intrinsic functional networks (Yeo et al., 2011). Figure 3A shows the correlation between FC and sgFC connection weights. As expected, the reweighing of FCs accentuates some connections and attenuates others. Supporting Information Figure S5 shows that long-distance connections tend to be stronger than expected, confirming the intuition developed in the previous subsection (Figure 2).

To investigate whether the reweighing of FCs reflects any organizational features of the brain, we first display FC and sgFC, now reordered by the canonical intrinsic networks (Figure 3B) (Yeo et al., 2011). To facilitate comparison, we standardize polysynaptic FCs by the overall average and standard deviation of FCs with direct SCs, which can be seen as FC informed by structure but not by geometry or distance. Interestingly, the largest differences between uncorrected and corrected FCs are observed within transmodal networks (default mode and ventral attention), while more modest differences are observed in the unimodal networks (visual and somatomotor) (Figure 3C). This suggests that unexpectedly strong FCs may occur more frequently between brain regions at the apex of the unimodal-transmodal cortical hierarchy. We investigate this possibility in the next section.

### Contribution to the Cortical Hierarchy

We next investigate the arrangement of unconnected FCs in macroscale cortical hierarchies. Recent work suggests that the functional architecture of human brain networks can be summarized by a small number of smooth topographic gradients, with the most prominent such gradient spanning unimodal to transmodal cortex (Margulies et al., 2016). This putative hierarchy is thought to support a sensory-fugal representational hierarchy (Mesulam, 1998) and correlates with spatial variation in cytoarchitecture (Paquola et al., 2019), myelination (Huntenburg et al., 2017), and gene expression (Burt et al., 2018).

**Figure 4.**
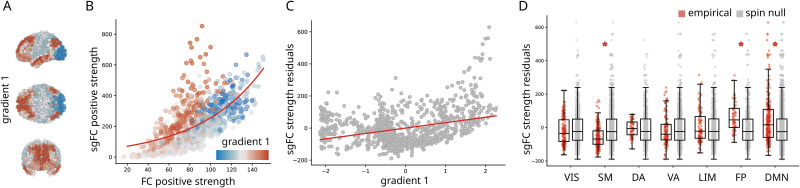
Contribution to the cortical hierarchy. (A) First principal connectivity gradient estimated using diffusion map embedding applied to the FC matrix (Margulies et al., 2016). Cold colors indicate unimodal regions and warmer colors indicate transmodal regions. (B) Correlation of positive strengths (sum of all weights incident on a given node) between sgFC and FC. Points are regions and are colored by their position in the unimodal-transmodal gradient. An exponential curve is fitted to the points (red line). (C) Residuals of the fitted curve in panel B correlated with gradient 1. (D) Residuals grouped by intrinsic networks and benchmarked against spatial autocorrelation-preserving null models (Alexander-Bloch et al., 2018; Markello & Misic, 2021). Statistically significant differences (with Bonferroni correction) are marked with a red asterisk.

To place each cortical node along this putative hierarchy, we adapted the diffusion embedding method described by Margulies and colleagues (Coifman et al., 2005; Margulies et al., 2016; Vázquez-Rodríguez et al., 2019) (see Materials and Methods for more detail). Figure 4A shows the topography of the first gradient, differentiating primary sensory and transmodal cortices, replicating the original report (Margulies et al., 2016).

To assess the hypothesis that unexpectedly strong FCs are more concentrated in transmodal cortex, we first compare node strengths (the sum of all weights incident on a given region) computed using FC and sgFC. Figure 4B shows the relationship between node strength for the original FC matrix and for the sgFC matrix. Nodes are coloured by their position in the hierarchy (gradient 1; red = transmodal, blue = unimodal). The relationship is well-fit by an exponential function (*y* = *e*^*x*^; *R*^2^ = 0.44). Importantly, a cloud of red points are consistent outliers, residing above the curve. In other words, brain regions at the apex of the hierarchy are more likely to participate in unexpectedly strong functional interactions.

We further confirm the link between the cortical hierarchy and unexpectedly strong FCs by computing the residual of each node relative to the exponential trend shown in Figure 4C (Pearson’s *r* = 0.34). Large positive residuals indicate that the node is disproportionately central in the sgFC functional network. Mean residuals for each intrinsic network, ordered by the unimodal-transmodal hierarchy, are shown in Figure 4D. The greatest increases appear in the fronto-parietal (*T* = 5.96, *p* = 1.26 × 10^−7^, *d* = 0.62) and default mode networks (*T* = 5.45, *p* = 1.13 × 10^−7^, *d* = 0.42), when compared to a null model that permutes region labels while preserving their spatial autocorrelation (Alexander-Bloch et al., 2018; Markello & Misic, 2021). Collectively, these results show that transmodal cortex participates in polysynaptic FCs that are stronger than expected given their length.

### Control Analyses

**Figure 5.**
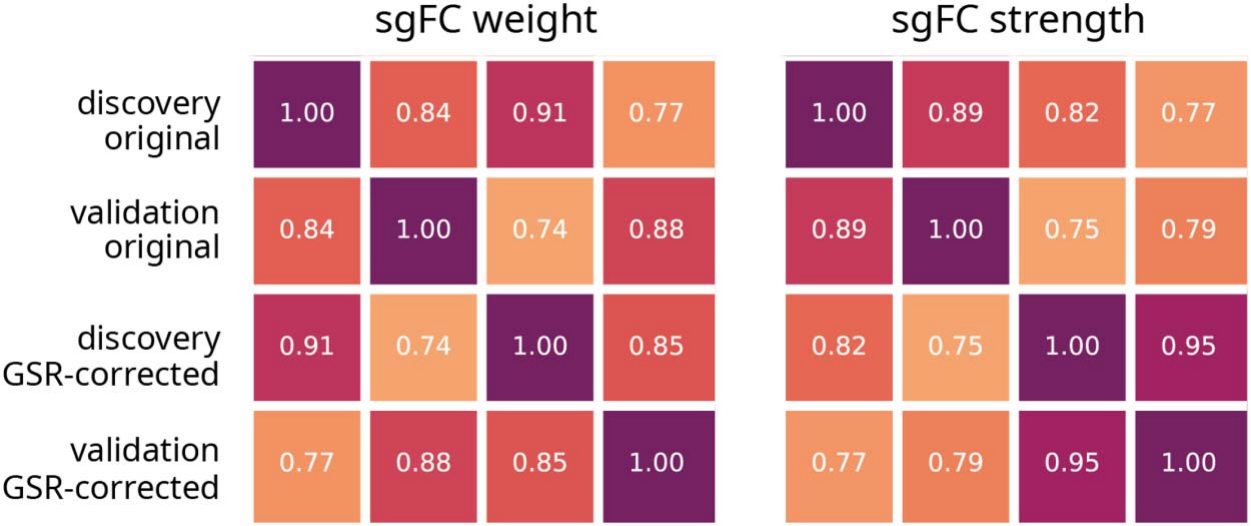
Validation and global signal removal. Correlation matrices shown for sgFC weight (values in Figure 2D) and sgFC positive node strength (values in Figure 4B) between controls. Values of sgFC are calculated from *Discovery* and *Validation* datasets, with and without global signal regression (GSR). Reproduced Figure 2D and Figure 4B with these values are shown in Supporting Information Figure S3.

The results presented in the preceding subsections are potentially contingent on a number of methodological choices, which we explore in detail here. We first replicate the major findings—the distribution of sgFC weights and their involvement in cortical hierarchies—in a validation cohort constructed from *N* = 33 participants. Figure 5 shows the Pearson correlation of the two results in the *Discovery* and *Validation* cohorts (see Supporting Information Figure S3 for reproduced result figures). The correlation coefficients for both measures are greater than 0.8 in all cases.

We next seek to determine the extent to which global signal regression could influence the findings. This particular preprocessing step induces negative correlations in FC, profoundly changing the distribution of weights (Aquino, Fulcher, Parkes, Sabaroedin, & Fornito, 2020; Murphy & Fox, 2017). We regenerated regional time series, correcting for fluctuations in the global signal, and repeated the analysis. Figure 5 shows the effects of the procedure, in both the *Discovery* and *Validation* cohorts (Supporting Information Figure S3). As before, there appears to be minimal change in the results, with correlations at approximately 0.9 (for weights) and 0.8 (for strength). In addition, correlations between data cohorts with different processing (e.g., *Discovery* set with no global signal regression correlated with *Validation* set with global signal regression) were also greater than 0.75.

## DISCUSSION

In the present report we introduce a simple data-driven method to benchmark functional connections with respect to their underlying structural and geometric embedding. We find evidence for unexpectedly strong functional connectivity among transmodal brain regions. These results suggest a hidden but highly organized pattern among polysnaptic FCs.

Our findings build on an emerging literature about the importance of geometry and structural connectivity for functional connectivity in the brain. Although the effect of spatial proximity on the probability and weight of connections is well known (Horvát et al., 2016; Roberts et al., 2016), in practice it is less obvious how this information should be taken into account when representing functional connectivity. Likewise, multiple studies report significant correlations between structural and functional connectivity between regions that share direct structural links (Honey et al., 2009), but how polysynaptic or multihop structural connectivity shapes functional connectivity is less well known. Indeed, computational models of structure-function coupling tend to perform more poorly when predicting functional connections between regions that are not structurally connected (Goñi et al., 2014). More recent communication models of structure-function coupling explicitly account for polysynaptic communication (Seguin, Tian, & Zalesky, 2020; Vázquez-Rodríguez, Liu, Hagmann, & Misic, 2020). Here we show that information about structural connectivity and spatial proximity can be naturally used as a frame of reference to describe functional connectivity between regions without direct structural connections.

Interestingly, we find that unexpectedly strong FCs are highly organized with respect to the modular (Sporns & Betzel, 2016) and hierarchical (Huntenburg et al., 2017) organization of the brain. Although both modules and hierarchies or “gradients” are robust and well-studied features of functional networks, their anatomical origin is less clear (Suárez et al., 2020). Our results suggest that both phenomena emerge from functional interactions or coactivations that transcend the underlying structure and geometry. In other words, this class of polysynaptic functional connections may be physiologically unique, and future empirical and theoretical studies could potential stratify direct and indirect FCs prior to analysis.

The fact that unexpectedly strong FCs are overrepresented in transmodal cortex may potentially explain recent reports that structure-function relationships are regionally heterogeneous. Namely, multiple reports have found that structure-function coupling is greater in unimodal cortex and smaller in transmodal cortex (Baum et al., 2020; Bazinet et al., 2021; Esfahlani, Faskowitz, Slack, Mišić, & Betzel, 2021; Gu, Jamison, Sabuncu, & Kuceyeski, 2021; Preti & Ville, 2019; Vázquez-Rodríguez et al., 2019; Wang et al., 2019). Our results suggest that the reason for this heterogeneity is that regions in transmodal cortex tend to participate in polysynaptic functional connections that are much stronger than expected given the underlying anatomical constraints. As a result, models relating structural and functional connectivity may be disadvantaged when applied to transmodal cortex relative to unimodal cortex.

More generally, the present framework is part of an emerging literature on simultaneously representing and modeling brain geometry, structure and function. A natural extension of sgFC is in the domain of spatially embedded null models that generate surrogate structural or functional networks to benchmark the presence of specific network attributes (Esfahlani, Bertolero, Bassett, & Betzel, 2020; Roberts et al., 2016). Moreover, sgFC may also serve as a quality function for generative models of connectivity (Akarca, Vértes, Bullmore, & Astle, 2021; Betzel et al., 2016; Oldham et al., 2021; Shinn et al., 2021; Vértes et al., 2012). Finally, we envision sgFC as the basis for more sophisticated network communication models that consider spatial proximity as a constraint for routing signals (Seguin, Razi, & Zalesky, 2019; Seguin, van den Heuvel, & Zalesky, 2018; Vázquez-Rodríguez et al., 2020). While these models traditionally focus only on spatial proximity, sgFC opens the possibility for a hybrid approach that takes into account structural connectivity and geometry.

The present results also need to be interpreted with respect to several methodological considerations. Despite the fact that we adopted a robust dataset and included a replication section, methodological choices including MRI acquisition scheme, processing pipeline, network reconstruction, and group consensus algorithm may still be susceptible to false positives and negatives (Jiang et al., 2021; Korhonen, Zanin, & Papo, 2021; Maier-Hein et al., 2017; Sarwar, Ramamohanarao, & Zalesky, 2021). In particular, the deterministic tractography procedure yields relatively sparse connectomes, and future work should consider the effect of connectome reconstruction and sparsity on the definition of polysynaptic FCs. In addition, systematic false positives or false negatives in connectome reconstruction could potentially emphasize or mask some classes of connections, such as long-distance connections. In this sense, the sgFC procedure developed here presents a framework to comprehensively compare multiple tractography pipelines with each other.

In summary, we show how fundamental structural and geometric priors can be used to reweigh and re-represent the functional connectivity matrix. Our results show that the canonical features of functional connectivity—modules and hierarchies—are delineated by unexpectedly strong functional connections between nodes without underlying structural links. The biological origin of this class of connections remains an exciting question for future research.

## MATERIALS AND METHODS

### Data Acquisition

A total of *N* = 66 healthy young adults (16 females, 25.3 ± 4.9 years old) were scanned at the Department of Radiology, University Hospital Center and University of Lausanne. The scans were performed in 3-Tesla MRI scanner (Trio, Siemens Medical, Germany) using a 32-channel head-coil. The protocol included (1) a magnetization-prepared rapid acquisition gradient echo (MPRAGE) sequence sensitive to white/gray matter contrast (1-mm in-plane resolution, 1.2-mm slice thickness), (2) a diffusion spectrum imaging (DSI) sequence (128 diffusion-weighted volumes and a single b0 volume, maximum b-value 8,000 s/mm^2^, 2.2 × 2.2 × 3.0 mm voxel size), and (3) a gradient echo EPI sequence sensitive to BOLD contrast (3.3-mm in-plane resolution and slice thickness with a 0.3-mm gap, TR 1,920 ms, resulting in 280 images per participant). Participants were not subject to any overt task demands during the fMRI scan.

### Structural Network Reconstruction

Gray matter was parcellated into 68 cortical nodes according to the Desikan–Killiany atlas (Desikan et al., 2006). These regions of interest were then further divided into four additional, increasingly finer grained resolutions, comprising 114, 219, 448, and 1,000 approximately equally-sized nodes (Cammoun et al., 2012). Structural connectivity was estimated for individual participants by using deterministic streamline tractography. The procedure was implemented in the Connectome Mapping Toolkit (Daducci et al., 2012), initiating 32 streamline propagations per diffusion direction for each white matter voxel.

To mitigate concerns about inconsistencies in reconstruction of individual participant connectomes (Jones, Knösche, & Turner, 2013; Thomas et al., 2014), as well as the sensitive dependence of network measures on false positives and false negatives (Zalesky et al., 2016), we adopted a group-consensus approach (Betzel et al., 2018; de Reus & van den Heuvel, 2013; Roberts, Perry, Roberts, Mitchell, & Breakspear, 2017). In constructing a consensus adjacency matrix, we sought to preserve (1) the density and (2) the edge length distribution of the individual participants matrices (Betzel et al., 2016, 2018; Mišić et al., 2015). We first collated the extant edges in the individual participant matrices and binned them according to length. The number of bins was determined heuristically, as the square root of the mean binary density across participants. The most frequently occurring edges were then selected for each bin. If the mean number of edges across participants in a particular bin is equal to *k*, we selected the *k* edges of that length that occur most frequently across participants. To ensure that interhemispheric edges are not under-represented, we carried out this procedure separately for inter- and intrahemispheric edges. The binary density for the final whole-brain matrix was around 2.1%.

### Functional Network Reconstruction

Functional MRI data were preprocessed using procedures designed to facilitate subsequent network exploration (Power, Barnes, Snyder, Schlaggar, & Petersen, 2012). fMRI volumes were corrected for physiological variables, including regression of white matter, cerebrospinal fluid, as well as motion (three translations and three rotations, estimated by rigid body coregistration). BOLD time series were then subjected to a low-pass filter (temporal Gaussian filter with full width half maximum equal to 1.92 s). The first four time points were excluded from subsequent analysis to allow the time series to stabilize. Motion “scrubbing” was performed as described by Power and colleagues (Power et al., 2012). The data were parcellated according to the same atlas used for structural networks (Cammoun et al., 2012). Individual functional connectivity matrices were defined as zero-lag Pearson correlation among the fMRI BOLD time series. A group-consensus functional connectivity matrix was estimated as the mean connectivity of pairwise connections across individuals.

### Structure- and Geometry-Informed Indirect FC Modeling

To construct the structure- and geometry-informed FC (sgFC), we apply equally spaced bins to the dimension of Euclidean distance. In each bin, we acquire the mean and standard deviation of those FCs with direct SC link. Then we take the z-score of FCs without direct SC link using the acquired statistics. The final z-scores are smoothed to get a robust representation by averaging over a spectrum of bin numbers (±25%) centering the optimal bin size decided by Freedman Diaconis Estimator shown in Supporting Information Figure S2. The resulting sgFC values corresponding to those without direct SC link are mapped back to a 1,000-by-1,000 matrix and used for network analysis through the article.

### Diffusion Map Embedding

Diffusion map embedding is a nonlinear dimensionality reduction algorithm (Coifman et al., 2005). The algorithm seeks to project a set of embeddings into a lower dimensional Euclidean space. Briefly, the similarity matrix among a set of points (in our case, the correlation matrix representing functional connectivity) is treated as a graph, and the goal of the procedure is to identify points that are proximal to one another on the graph. In other words, two points are close together if there are many relatively short paths connecting them. A diffusion operator, representing an ergodic Markov chain on the network, is formed by taking the normalized graph Laplacian of the matrix. The new coordinate space is described by the eigenvectors of the diffusion operator. We set the diffusion rate *α* = 1 and the variance of the Gaussian used in affinity computation *σ* = 1. The procedure was implemented using the Dimensionality Reduction Toolbox (https://lvdmaaten.github.io/drtoolbox/) (Van Der Maaten, Postma, & Van den Herik, 2009).

## ACKNOWLEDGMENTS

We thank Justine Hansen, Vincent Bazinet, Golia Shafiei, Estefany Suarez, Andrea Luppi, and Filip Milisav for their comments and suggestions on the manuscript.

## SUPPORTING INFORMATION

Supporting information for this article is available at https://doi.org/10.1162/netn_a_00236.

## AUTHOR CONTRIBUTIONS

Zhen-Qi Liu: Conceptualization; Formal analysis; Methodology; Visualization; Writing—Original draft. Richard F. Betzel: Formal analysis; Writing—Review & editing. Bratislav Misic: Conceptualization; Methodology; Supervision; Writing—Original draft; Writing—Review & editing.

## FUNDING INFORMATION

Bratislav Misic, Canada First Research Excellence Fund (https://dx.doi.org/10.13039/501100010785). Bratislav Misic, Natural Sciences and Engineering Research Council of Canada (NSERC Discovery Grant), Award ID: 017-04265. Bratislav Misic, Canada Research Chairs (https://dx.doi.org/10.13039/501100001804). Award ID: SFB 936/Z3.

## Supplementary Material



## TECHNICAL TERMS

Structural connectivity: Anatomical projections between neural elements.
Functional connectivity: Temporal coactivation among neural elements.
Monosynaptic: Communication between neural elements via a direct anatomical projection.
Polysynaptic: Communication between neural elements via a sequence of multiple anatomical projections and intermediate neural elements.
Intrinsic networks: Subnetworks of brain areas with coherent time courses, identified by clustering, independent component analysis, or community detection.
Cortical hierarchy: A continuous axis separating unimodal sensory-motor cortex from transmodal association cortex.
Global signal: The mean time course computed over all voxels within the brain.
