# Supplementary material for: Benchmarking functional connectivity by the structure and geometry of the human brain
Source: Netw Neurosci. 2022 Oct 1;6(4):937–49. doi: 10.1162/netn_a_00236 (PMC9976650; doi:10.1162/netn_a_00236)
Supplement: Supplementary file 1 [file netn-06-937-s001.pdf]

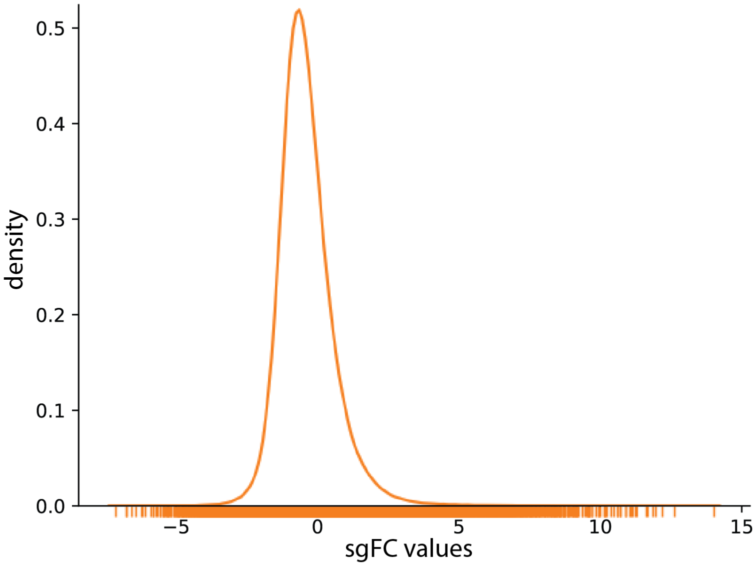

Figure S1 | **sgFC value distribution.** Distribution of sgFC values is shown as both a kernel-estimated probability density and individual points.

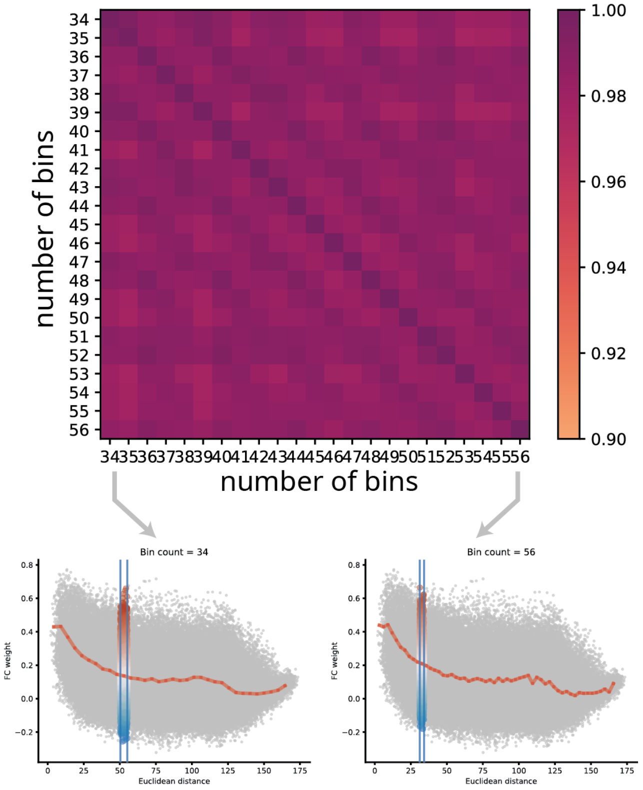

Figure S2 | **Influence of bin choice.** Correlation matrix shown for sgFC values between choices of bin numbers. The number of bins are taken $\pm 25\%$ centering the optimal bin size decided by Freedman Diaconis Estimator. The final sgFC values are averaged over the choices of bin numbers to get a smoothed robust representation.

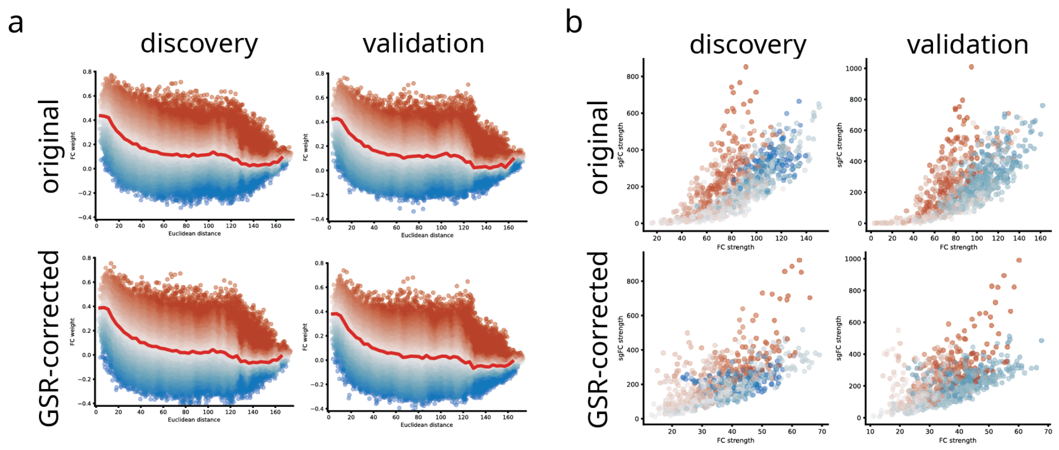

Figure S3 | **Results of control analyses.** Reproduced Fig. 2d and Fig. 4b under control analyses settings. Correlations between the control cases for values in panel a (sgFC weight) and panel b (sgFC node strength) are shown in Fig. 5.

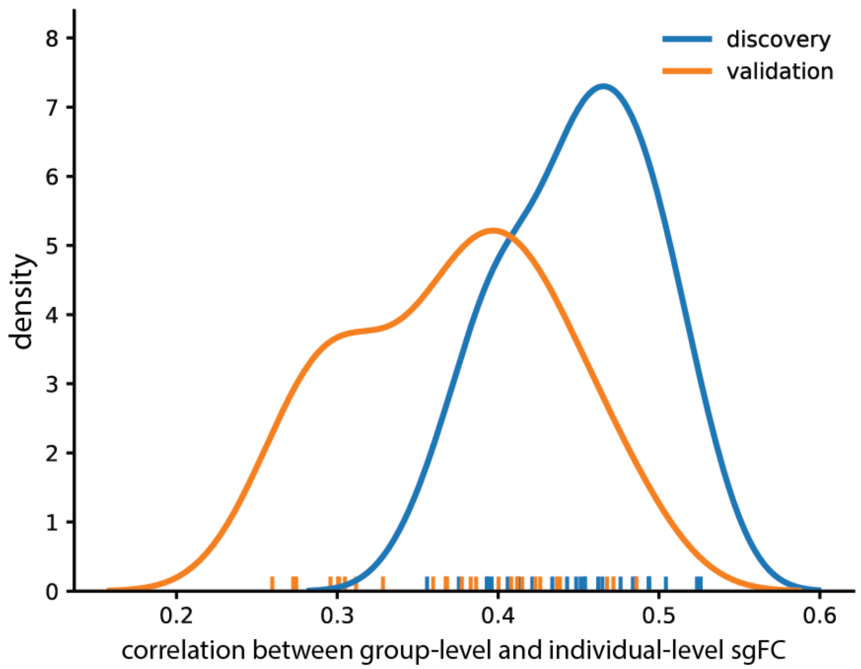

Figure S4 | **sgFC in individual participants.** Distribution of correlation between group-level sgFC used in the main analysis and those for $N = 66$ participants. Group-level sgFC values for the *Discovery* dataset are correlated with sgFC generated from each participant in the *Discovery* and *Validation* dataset.

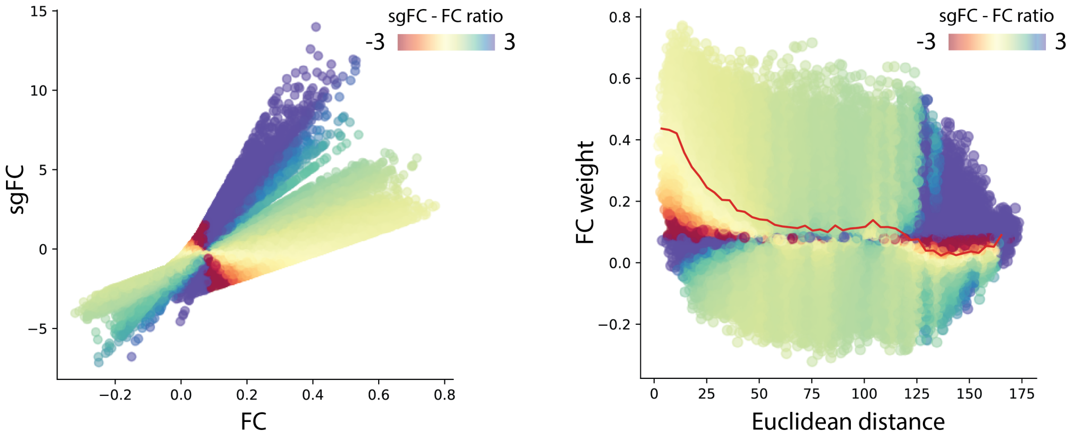

Figure S5 | **Ratio of sgFC to FC** The z-score(sgFC) to z-score(FC) ratios are calculated from Fig. 3a data points, and displayed for Fig. 3a (left) and Fig. 2d (right), roughly delineating the “slope groups”.
